# Only severe Injuries are effectively reduced by muscles' injury prevention protocols in football players: A systematic review

**DOI:** 10.12688/f1000research.148925.1

**Published:** 2024-06-26

**Authors:** Rihab Moncer, Marwa Ghanmi, Dhekra Chebil, Sana Bhiri, Iheb Belhadj Youssef, Amani Kacem, Sana Salah, Sahbi Mtaouaa, Sinen Frigui, walid Ouannes, Sonia Jemni

**Affiliations:** 1 Physical and rehabilitation department, Sahloul Hospital of Sousse, Tunisia, Faculty of medicine of Sousse, University of Sousse, Tunisia, Sousse, 4054, Tunisia; 2 Department of physical and rehabilitation, Kairouan, Tunisia, Faculty of medicine of Sousse, University of Sousse, Tunisia, Kairouan, Tunisia; 3 Hygiene department, Kairouan, Faculty of medicine of Sousse, University of Sousse.Tunisia, Kairouan, Tunisia; 4 Hygiene department, Sahloul Hospital of Sousse, Faculty of medicine of Sousse, University of Sousse, Tunisia, Sousse, 4054, Tunisia, Sousse, Tunisia; 5 physical and rehabilitation medicine Ksar Hlel, Monastir, Faculty of medicine of Monastir. University of Monastir. Tunisia, Monastir, Tunisia; 6 Department of pneumology Kairouan, Faculty of medicine of Sousse, University of Sousse, Tunisia, Kairouan, Tunisia; 7 Department of physical and rehabilitation, Fattouma Bourguiba hospital, Faculty of medicine of Monastir. University of Monastir. Tunisia, Monastir, Tunisia

**Keywords:** muscle, wounds and injuries, prevention, exercise, football, soccer, systematic review

## Abstract

**Background:**

Muscle injuries are the most frequent in football and may lead to the end of a player’s career. Many studies have evaluated the effectiveness of prevention programs for all injury types. Few studies have evaluated the effects of exercise on muscle injuries.

**Methods:**

A documentary search was conducted in October 2022 from three databases: Medline via its PubMed interface, Google Scholar, and COCHRANE databases. We only included original articles published between October 2012 and October 2022 with a high level of evidence. The research was conducted according to the Preferred Reporting Items for Systematic Reviews and Meta-Analyses criteria. The target population consisted of professional and semi-professional footballers. The intervention in question was structured injury preventive protocols, including specific training or pre-established protocols such as the FIFA 11+ program, compared to each other or with regular training sessions. The main outcomes were the incidence rates of muscle injuries and severe injury in each group, as well as the time loss to injury.

**Results:**

Seven randomized controlled trials with a high level of evidence confirmed using the PEDRO scale were included. There were 3815 male professional football players. There was heterogeneity in the age, hours of exposure, and duration of the intervention. The prevention protocols used were FIFA 11+, bounding exercises, NHE, and stretching exercises. Structured exercises significantly reduce the severity of muscle injury and, consequently, time loss to injury.

**Discussion:**

Despite the diversity of prevention programs, teams still lack standardized programs. Structured exercises, such as FIFA 11+ and bounding exercises, which combine strengthening, body kinesthesic awareness, and neuromuscular control, reduce the incidence and severity of muscle injury. FIFA 11+ is the first and most complete structured program, but lacks some exercises, such as adductor strengthening.

**Conclusion:**

This review showed the interest in structured exercises in reducing severity and time loss to injury in professional football players.

**Registration:**

This review was registered on 8/17/2023 in the International Prospective Register of Systematic Reviews under the reference code CRD42023452202.

## Introduction

Muscle injury (MI) is the most common injury in football. ^1^ The incidence of total injuries varies between 1.06/1000 hours (hours) and 5.87/1000 hours of training and competition. ^2^ MI is typically caused by repeated eccentric muscular contractions. ^3^ The relationship between eccentric muscular contraction and MI is confirmed by the lower occurrence of MI during cycling and swimming, two sports based almost exclusively on concentric muscle contraction. ^4^^,^^5^ Hamstring muscles are the most frequent site of MI in professional football, where they cause the highest number of absence days during the sporting season (25% of all absences due to injuries), with an average recovery time of nine days. ^6^^,^^7^

Eccentric strengthening is considered effective in the prevention of MI because it results from an eccentric strength deficit in comparison with the concentric force of hip extensors in elite sprinters. ^6^ Among sports science researchers who aimed to develop a global and universal program for injury prevention, F-MARC conducted the first study on exercise-based prevention of football injuries, showing 21% fewer injuries in the intervention group (IG) than in the control group (CG). ^7^ The interventions focused on improving the structure and content of the training by educating and supervising coaches and players. The program included preventive interventions, such as improvement of warm-up, regular cool-down, taping of unstable ankles, adequate rehabilitation, promotion of the spirit of fair play, and 10 sets of exercises. It is designed to improve coordination, stability of the ankle and knee, and flexibility and strength of the trunk, hip, and leg muscles. Subsequently, F MARC, in cooperation with international experts developed the FIFA11 (Federation Internationale Football Association). ^8^

Evidence of the efficacy of this program has been reported in several studies. ^9^^,^^10^ Many trainers have judged that it is a lengthy program and that players find it difficult to adhere to it, given the absence of exercises involving the ball. ^9^ Therefore, modifications were added; hence, FIFA11+. ^11^

For more than 10 years, researchers and researchers have been performing exercises to identify the most effective ones in MI prevention. Knowing the most effective program with evidence-based practice will help improve prevention measures and reduce the incidence of MI in football.

### Objective

The objective of this study is to report, through a systematic review of current scientific research of high level of proof (randomized controlled trial) the efficacy of programs for the prevention of muscle injuries for football players.

## Method

The present research work is a systematic review of the biomedical literature to provide an analysis of the preventive effect of programs for muscle injuries in football. A documentary search was conducted in December 2022 from three databases: Medline via its PubMed interface, Google Scholar, and COCHRANE databases, through the following documentary request: Which exercises are effective in MI in professional football players?

The MESH terms used were prevention, muscle, injury, wounds and injury, soccer, and exercise.

The combinations of these different keywords generated the following search equation:

“Prevention” OR “Prévention” AND “muscle” AND “Wounds and injury” OR “plaies et blessures” AND “Exercise” OR “Exercice” AND “Football” OR “SOCCER”

The research question was methodically put in the PICO format to identify appropriate keywords. After exploring the digital databases, we studied the bibliographical references of relevant articles.

We included an independent double lecture of the title/abstract and full texts by two trained specialists in rehabilitation and physiotherapy (R. M) and preventive and health public medicine (D. CH), and studies on the preventive effect of program or exercise on MI.

I- **SOURCES OF INFORMATION AND SEARCH**
  1. COMPUTER RESEARCH: We used scientific search engines: PUBMED, Google Scholar and COCHRANE database.
  2. MANUAL RESEARCH: After exploring the digital databases, we studied the bibliographical references of the articles deemed relevant.
II- **ELIGIBILITY CRITERIA**
  1. **CHARACTERISTICS OF THE STUDY**
Our question was methodically put in the PICO format:
    ○ **POPULATION**
    ○ Professional and semi-professional male and female footballers
    ○ Amateurs were excluded due to a lack of dedicated physiotherapy staff on their football teams and the lower time of training and playing compared to their professional counterparts.
We also excluded Gaellic, Futsal, Australian and American Football players from the study.
Children were also excluded because they did not have the same number of hours playing and the same type and frequency of injury.
    ○ **INTERVENTIONS**
Application of structured muscle injury preventive protocols, including specific training, physical therapy, or pre-established protocols such as the FIFA 11+ program, aimed at reducing the risk of MI.
    ○ **COMPARISONS**
We included controlled studies that compared different protocols and regular training sessions with an acceptable number of hours of exposure to training and competitions.
    ○ **OUTCOMES**
IR (Incidence Rate) of injuries and severe injuries per 1000 h (Injury/1000 h) of exposure and time loss to injury, including training and official competitions.
  2. **INCLUSION CRITERIA**
We included original articles published between December 2012 and October 2022 available in digital versions, written in French or English with a high level of evidence (Level I and II), which evaluated the efficacy of the prevention program on muscle injury with at least 2 months of follow-up, monitoring players between training and competition.
  3. **EXCLUSION CRITERIA**
We excluded:
Studies of the meta-analysis or literature review type: These two types of publications were excluded from our research to avoid duplicating the results of the clinical trials, which may also be included in these reviews.
    ○ Communications in congresses, consensus conferences of learned societies.
    ○ Studies that did not correspond to research quotes (adjustment, technical notes, letters to the editor, case reports, interviews, opinions, single commentaries, etc.).
    ○ Works not available for consultation or only available in paper format.
Articles with less than two months of follow-up or with too few total hours of exposure between training and competition were excluded.
Studies that assessed MI in amateur footballers were not included because the risk of injury in amateur teams is much lower than that in professional clubs, which would considerably increase the risk of bias in statistical analysis.
    ○ Studies that assess the adherence to a prevention program.
    ○ Studies that have studied the effect of exercise on performance and on weight joint injuries, central pivot.
    ○ Articles that study the effect of exercise on performance, sleep, psychology
III- **SEARCH STRATEGY**
  The study was conducted following the guidelines for writing a literature review according to the PRISMA criteria (Preferred Reporting Items for Systematic Review and Meta-Analyses). ^12^ It was registered in the International Prospective Register of Systematic Reviews (PROSPERO) under the reference code CRD42023452202. The total number of identified articles was documented based on the search strategy. The references of the articles were recorded using the Zotero https://www.zotero.org/download bibliographic software https://www.zotero.org/download to sort the articles and eliminate duplicates.
IV- **SORTING AND SELECTION OF ARTICLES**
  For article selection, the reviewers screened the titles and/or abstracts. Duplicates and articles were removed first. If the title and/or abstract were not sufficient for inclusion or exclusion, the full-text article was retrieved and read independently by two reviewers (R. M and D. CH). In addition, a cross-reference search of the selected articles was performed to identify agreement and differences. If consensus could not be reached among the investigators regarding the inclusion or exclusion of an article, a third reviewer (S. B.) was consulted to make the final decision.
  Finally, the two assessors completed data extraction independently.
  The selected articles were sorted according to their general characteristics (main author, journal where the study was published, year of publication, country where the work was carried out, type of study, and level of proof ).
V- **Quality of article assessment and risk of bias**
  Given the heterogeneity between the study design and methodology adopted, the rating scale Physiotherapy Evidence Database (Pedro) scale, available online, ^13^ was used to critically appraise the quality of the studies selected and retained in the present systematic review.
  The PEDro scale was used to rate each article and inform clinicians of its degree of quality. The scale also assessed the risk of bias of the studies in its items (randomization, allocation concealed, blinding subject, blinding therapist, and blinding assessors). The last four items of this scale evaluate the measurement bias of the studies. A Pedro scale score higher than 6/10 had a low risk of bias.
VI- **Data extraction and synthesis**
  Following the critical appraisal, relevant data were systematically extracted and tabulated from the included studies. Extracted data included the study title, year, authors, study type, population characteristics, description of exercises applied by researchers, main findings of the studies, and methodological quality assessment scores.
  The main findings were as follows: the difference between the IG and CG according to their injury rate (IR), severity of injuries/1000h of play, and time loss due to injury. All outcomes were analyzed using a critical narrative synthesis.

## Results

**Figure 1.**
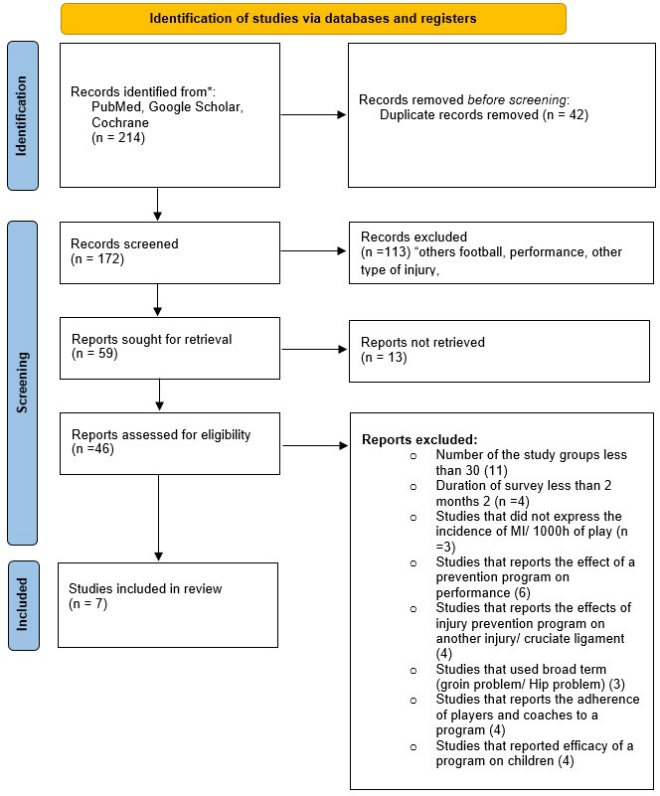
PRISMA flowchart of the study.

A total of 214 articles, with 112 articles from Medline, 31 articles from GOOGLE SCHOLAR, and 71 articles from the Cochrane library, were initially identified. Of these, 42 duplicates were excluded. The remaining 172 articles were screened against the eligibility criteria by title and abstract to exclude those clearly not relevant to the review topic. Following this, the full texts of 59 articles of potential relevance were retrieved, and 39 were excluded based on the eligibility criteria. Once the screening and selection processes were complete, seven articles were eligible for inclusion in the present systematic review. ( Figure 1)

### I- General characteristics of the studies

All the studies included were cluster randomized controlled studies, except the study by Azuma et al., which was a randomized controlled trial [ 14] ( Table I).

**Table I.** General characteristics of the included studies.

Author’s Article	Journal	Journal Rating IF	Year of publication	Country	Type of the study	Level of evidence
Azuma et al [ 14]	Scand J Med Sci Sports	4,65 Q1	2019	Netherlands	cluster-randomized controlled trial	I
Hammes et al [ 15]	Scand J Med Sci Sports	4,65 Q1	2020	Japan	Randomized controlled trial	I
Granelli et al [ 16]	Journal of sports sciences	Q1	2014	Switzerland Germany	cluster-randomized controlled trial	I
Owoeye et al [ 17]	AmJSportsMed	Q1	2015	USA	cluster-randomized controlled trial	I
Whalan et al [ 18]	Journal of sports sciences & medicine	4,01 Q1	2014	NIGERIA	cluster-randomized controlled trial	I
Van de Hoef [ 19]	Scand J Med & Sci Sports	4,65 Q1	2017	Australia	Cluster-randomised controlled trial	I
Hasebe et al [ 20]	International journal of sports medicine	Q1	2020	Japan	cluster-randomized controlled trial	I

### II- Quality assessment of the studies

The included studies specified the eligibility criteria and concealed allocations. In addition, the participants were randomized into similar groups in all studies. However, none of them blinded the participants, conductor, or examiner, except for Whalan et al. The results were adequately reported for injury rates in all the studies. The mean PEDRO scale of all included studies was > five. Thus, the average quality of the included studies was satisfactory ( table II)

**Table II.** Quality and biais assessment of selected article by Pedro scale.

Pedro scale	Van De Hoef et al [ 19]	Azuma et Someya [ 14]	Hammes et al [ 15]	Granelli et al [ 16]	Owoeye et al [ 17]	Whalan & al [ 18]	Hasebe &al [ 20]
1- Eligibility criteria specified	**+**	**+**	**+**	**+**	**+**	**+**	**+**
2- Randomization	**+**	**+**	**+**	**+**	**+**	**+**	**+**
3- Allocation concealed	**+**	**+**	**+**	**+**	**+**	**+**	**+**
4- Groups similar	**+**	**+**	**+**	**+**	**+**	**+**	**+**
5- Blinding all subjects	**_**	**_**	**_**	**_**	**_**	**_**	**_**
6- Blinding therapists	**_**	**_**	**_**	**_**	**_**	**_**	**_**
7- Blinding assessors	**_**	**_**	**_**	**_**	**_**	**+**	**_**
8- Measures of at least one key outcome were obtained from more than 85% of the subjects initially allocated to groups	**+**	**+**	**+**	**+**	**+**	**+**	**+**
9- All subjects for whom outcome measures were available received the treatment or control condition as allocated or, where this was not the case, data for at least one key outcome was analysed by “intention to treat”	**+**	**_**	**_**	**_**	**_**	**_**	**_**
10- The results of between-group statistical comparisons are reported for at least on key outcome	**+**	**+**	**+**	**+**	**+**	**+**	**+**
11- The study provides both point measures and measures of variability for at least one key outcome	**+**	**+**	**+**	**+**	**+**	**+**	**+**
12- Score PEDRO/10	**7**	**6**	**6**	**6**	**6**	**7**	**6**

+: done.

-: not done.

### III- Studies’ populations

A total of 3815 players were included in the seven studies, and all included participants were Males. The characteristics of mean age, BMI, and total exposure time are presented in Table III.

**Table III.** Studies’ populations.

Study		IG	CG
Van de Hoef et al [ 19]	Age	23.8 ± 6.4	22.2 ± 3.1
	Height	183.0 ± 8.6	182.6 ± 6.6
	weight	78.88 ± 8.6	76.15 ± 7.4
	Total exposure (h)	139.3	127.0
Azuma et al [ 14]	Age	16.2±0.8	16.2±0.8
	BMI	21.1±1.4	20.9±1.2
	Total exposure (h)	121.0±12.3	122.3±12.2
Hammes et al [ 15]	Age	45.2 (7.7)	43.1 (6.5)
	BMI	27.0 (3.4)	26.1 (2.7)
	Total exposure h	4172	2937
Granelli et al [ 16]	Age	20.40 ± 1.66	20.68 ± 1.46
	BMI	**NM**	**NM**
	Total exposure/1000h	35226	44212
Owoeye et al [ 17]	Age	17.80 (.94)	17.49 (1.10)
	BMI	21.82 (1.65)	21.22 (1.46)
	Total exposure h	51,017 (5,101.7 ± 1473.7)	61,045 (6,104.6± 2,976.5)
Whalan et al [ 18]	Age	24.8 [24.0,25.6]	23.8 [23.0,24.7]
	Height	176.9 [176.2,177.6]	178.3 [177.5,179.1]
	Weight	79.3 [78.9,79.7]	78.3 [77.9,78.7]
	Total exposure h	26062.1	28541.4
Hasebe et al [ 20]	Age	16.7 ± 0.5 (15–18)	16.3 ± 0.6 (15–17)
	Height	171.0 ± 5.0 (155.0–188.0)	171.0 ± 5.3 (160.0–183.0)
	Weight	61.4 ± 5.7 (48.0–76.0)	61.5 ± 5.4 (50.0–75.0)
	Total exposure h	1055	855

IG: Interventional group;

CG: control group;

NM: not mentioned

### IV- Prevention protocol

The seven included studies were cluster randomized to avoid contamination within the same team, except for the study by Azuma et al. ^14^

Several programs have been proposed in these studies. There was heterogeneity in the intervention duration.

1. **1- Fifa11+**
  In a total of 265 players included, Hammes et al. conducted a cluster randomized study where they proposed FIFA 11+ for 119 IG and 146 CG for 9 months. ^15^
  In addition, in the study by Granelli et al., 675 players performed FIFA11+ compared to the usual training session performed by 850 players for 8 months. ^16^
  Owoeye et al. 212 performed FIFA11+ and 204 usual training sessions for 6 months. ^17^
  Whalan et al. suggested another type of control between groups: for CG 408 players, a modified FIFA 11+, in which “Part 2” of the program was performed at the end of training, as opposed to “Part 1”and “Part 3,,” which were performed at the beginning. In the IG, 398 players performed FIFA 11+. ^18^
2. **2- BEP**
  For 400 players, Van de Heuf et al. proposed BEP (concentric to eccentric than plyometric exercises) for 229 players in IG and 171 usual training sessions in CG for a period of 12 weeks. ^19^
3. **3- NHE**
  Hasebe et al. suggested NHE for 156 players in the CG and usual training for 103 players in the CG for 27 weeks. ^20^
4. **4- Stretching**
  For 40 weeks, Azuma et al. conducted a stretching protocol for 64 IG players. Physical therapists provided personal exercise instructions three times a week. The program was made of 10 stretching exercises and didn’t require the use of any equipment. Tight body parts were held for 30 s with a rest interval of approximately 30 seconds. Similar exercise instructions were provided during the cooling period. The players were permitted to continue the exercise program themselves after the intervention period. Players in the CG performed the usual training session. ^14^

### V- Main outcomes

All studies have shown that structured programs reduce MI rates, but significant differences have been observed, especially in the severity of injuries.

The bounding exercise applied in the IG in the study by Van de Heof et al. did not significantly reduce IR or severity of injury. ^19^

FIFA 11+ used in the studies of ^15^^–^^18^ showed significant differences in severe injuries in most cases.

In the study by Azuma and Someya, the stretching protocol significantly reduced IR and severe injury. ^14^

The NHE reduced IR without a significant difference in the study by Hasebe et al. ^20^ ( Table IV)

**Table IV.** Detailed interventions performed in the studies and their main outcomes.

Research Paper	Intervention group	Control group	Duration of intervention	Key findings IR (/1000h of exposure) Statistic difference between both groups
Van de Hoef et al [ 19]	**N= 229** build-up program ( **concentric to eccentric to plyometric exercises**) and a maintenance program that takes approximately 30-50 minutes to complete	**N= 171** Usual soccer training	**12 weeks**	IG= 1.12 vs CG= 1.39 No significant difference between the IG and CG in HI during the season (OR = 0.89, 95% CI 0.46-1.75) Mean number of days off play: 33.0 ± 42.7 in the IG Vs 21.35 ± 12.7 in the CG No significant difference in time-to-first HI (HR = 0.90, 95% CI = 0.48-1.70) No statically difference in severity of injury ( **P =0,48**)
Azuma & Someya [ 14]	**N= 64** Personal exercise instructions 3 times a week. 10 stretching exercises, held for 30 seconds. Rest interval about 30 seconds. Similar exercise instructions, during cool-down.	**N=60** Usual training without stretching	**40 weeks** **“12 supervised** **28 done by the player”**	IG =2,67 vs CG=3,41 (p <0.01)* Rate Ratio: 0.37(0.20-0.66) Significant difference in severe injury: IG= 0.2 Vs CG=0.99 (p<001)*
Hammes et al [ 15]	N=119 FIFA 11 + training session	N=146 Usual training session	**9 months**	**IG= 7.1** Vs **CG** =6.5 (P 0.49) significant difference in severe injuries: **CG (**5.8 **)** and **IG** (2.6) (IRR 0.46 [0.21–0.97] P = 0.04* Mean number of days off play: 17. IG: 14 (10/25) and CG: 27 (12/39); P = 0.04*
Granelli et al [ 16]	N= 675 FIFA 11+	N= 850 Usual training session	**8 months**	HIR I G =0.454 Vs CG =1.22 Rate Ratio (IC 95%)= 0.37 (0.21-0.63) QIR: CG 0.99 Vs I G 0.71 RR (IC 95%)= 0.72 (0.44-1,16) Mean time of loss to injury (days): CG (13.20 ± 26.6) Vs IG (10.08 ± 14.68) (p= 0.007)*
Owoeye et al [ 17]	**N= 212** FIFA 11+	**N=204** Usual training session	**6 months**	IR **CG** 0.2 Vs **IG** 0.0 Rate ratio (IC 95%) = 0.19 (0.04-1.01) P=0.052
Hasebe et al [ 18]	**N=156** Nordic hamstring exercise	**N=103** Usual training session	**27weeks**	HIR 1.04 CG Vs 0.88 IG Rate Ratio: 1.14 Mean time of loss to injury: 1116.3/10000h in the CG and 113.7/10000h in the IG with RR 9.81
Whalan et al [ 20]	**398** Entire 11+ before training twice per week Parts 1 and 3 before matches	**408** Part 1+3 before training Part 2 after training	**All the 2017 season**	IR: 5,2 IG VS 4,9 the CG (P=0,88) HIR: IG 2 Vs 1,6 CG (P=0,37) QIR: IG 0,7 Vs 1,2 in CG(P= 0,09) Total time loss of all types of injury (days): 4303 CG vs 5815 IG injuries with P=0,026*

CG: control group;

HIR: hamstring injury rate;

IG: intervention group;

IR: injury rate;

RR: relative risk;

QIR: Quadriceps injury rate.

## Discussion

Despite the existence of multiple effective programs, teams still lack standardization in terms of MI prevention programs. This review aimed to identify the most effective programs for the prevention of MI in football. From a global view, structured programs reduce the MI rate, but more significantly, its severity and time loss to injury.

Previously, many reviews were interested in either evaluating the efficacy of one particular program ^10^ or the efficacy of programs on one particular group of muscles. ^21^^–^^22^ In the hamstrings review, ^21^ there was not a sufficient number of participants included in the studies (less than 20), and only the study by Whalan et al., which was included in our review, included more than 30 participants.

FIFA 11+ is the most commonly used MI prevention program. ^15^^–^^18^ FIFA 11+ is a comprehensive warm-up program aimed at improving muscular strength, body kinesthetic awareness, and neuromuscular control during static and dynamic movements. This program consists of three parts, 15 different exercises with a focus on neuromuscular effects on the lower extremities and core. The first and third parts include running exercises; it begins “at slow speed combined with active stretching and trolled partner contacts” and finishes “at moderate/high speed combined with planting/cutting movements”. These parts include the dynamic actions and accelerations. Part 2 consisted of six strength, plyometric, and balance exercises with a focus on core and leg strength, proprioception, and stability. Three variation levels were provided for each exercise. The 11+ program was designed to be delivered as a 20-25 min “warm-up” before commencing other training activities, without the need for specialized equipment. ^9^ Its effectiveness has not been fully documented in the literature; Steffen et al., in 2013, reported an overall significant injury reduction of 72% based on secondary data analysis. ^23^

There were only significant differences in the study by Hammes et al. ^15^ in terms of IR between the IG and CG. In the other studies included, FIFA 11+ did not show a reduction in IR, but there was a significant difference in terms of the severity of injury.

All players in the included studies were male, but FIFA 11+ was tested several times in female soccer players ^24^^–^^25^; however, the number of participants was not enough to conclude efficacy.

In the study of Mckey et al, ^26^ authors evaluated the impact of high versus low application of FIFA 11+ and found significant difference in the study of High application of FIFA 11+ but the number of participants was few “23 females” to consider those findings. ^26^

From the seven studies included, Whalan et al. ^18^ used a particular control within group; for IG, they applied classic FIFA 11+ described above, and for CG, they proposed parts 1 and 3 before training and part 2 after training. However, significantly fewer severe injuries and days lost to injury were observed in the “P2 post” group in this study. The authors explain these findings by better compliance and adherence in the P2 post-training group, which provides a simple and practical method to potentially improve the effectiveness of the 11+ program and compliance with the 11+ program. ^18^

Indeed, implementation of FIFA 11+ was initially difficult, and there was a lot of resistance to adherence from both coaches and players. ^11^^,^^23^^,^^25^^,^^26^ Many authors have studied the factors influencing adherence to the FIFA 11+ program and identified barriers to its implementation, such as program duration and exercise difficulty/fatigue. They have also shown that this adherence may be related not only to the effectiveness of the program itself, but also to beliefs and practice in football coaches’ attitudes. ^26^

In addition to FIFA 11+, Vand de Hoef used a complete bounding exercise programme. Players perform a gradual build-up of walking lunges, tripling and drop lunges, and bounding (alternating leg jumps). ^19^

After a period of 12 weeks of buildup, players will continue BEP for the rest of the season, two sessions a week. The first gradual build-up should improve functional movement patterns and increase strength. ^27^

The bounding exercise, in the literature referred to ‘a running bound’ or ‘alternate leg bounding’, is a popular running-specific exercise that can be categorized as plyometric exercise. These plyometric exercises consist of three phases: eccentric pre-stretch, amortization phase (time between eccentric and concentric contractions), and concentric shortening phase. ^28^

Other studies focused only on a group of muscles. For example, Hasebe et al. studied the effectiveness of NHE on hamstring injury (HI). ^20^ The athletes were asked to use their arms and hands to buffer the fall, let their chest touch the surface, and return immediately to the starting position by pushing with their hands to minimize loading in the concentric phase. In this study, Hasebe et al. showed that NHE might not significantly reduce IR; however, it significantly reduced the time lost to sports due to injury (p=001).

According to literature, HI is the most frequent MI and injury in soccer. In 2001, Mjolsnes introduced the NHE, presented as an exercise that can easily be performed on pitch without special equipment, and in a randomized controlled study, the NHE group presented a 57% lower HSI (Hamstring Injury). ^29^

In addition, in a recent systematic review and meta-analysis of six studies, BIZ et al. concluded that both NHE and FIFA11+ are efficient for HSI. ^21^

In the study by Azuma and Someya, ^14^ the beneficial effect of stretching intervention was particularly strong for severe injuries that require a long time to return to play. Stretching is regularly included in warm-up exercises. However, some authors have reported the opposite finding for stretching. ^30^^,^^31^ The effects of stretching on MI depend on the type of sport. In sports with a high stretch-shortening cycle, such as football, when a compliant muscle-tendon unit is contracted, most of the energy is absorbed by the tendon and then MI is reduced. However, in other types of sports with low or no stretch-shortening cycles, such as cycling, most athletes are used to stiff tendons, and stretching will ll not directly cause MI. In fact, having a more compliant tendon makes them less adapted to their sports activities, so they develop more injuries. ^30^^,^^32^

Therefore, we can conclude that stretching is recommended for MI in soccer.

We did not include studies that used a specific program for adductors, which are frequently injured in football, because they did not meet our inclusion criteria. Most of the studies used the Copenhagen adduction exercise (CA). ^33^^–^^35^ It is a partner exercise. The player lies on the side with one forearm supporting him on the floor and the other arm placed along the body. The upper leg is held at approximately the hip height of the partner, who holds the leg using one arm and supports the knee with the other. The player then raises his or her body from the field, and adducts the lower leg so that the feet touch each other, and the body is in a straight line. The body was then lowered halfway to the ground while the foot of the lower leg was lowered so that it touched the floor without being used for support. This exercise was performed on both sides. It is an eccentric hip adductor exercise that prevents adductor injury. Haroy et al. showed that adding this type of exercise to FIFA 11+ may compensate for the lack of eccentric hip strengthening exercise. ^35^ In addition, Alonso et al. ^34^ showed that CA increased muscle thickness, which may reduce adductor injury. ^34^

As there are few players to conclude the effectiveness of this program, more studies with more players should be conducted for specific adductor prevention programs.

The most relevant point of our study was the diversity of programs used to prevent MI in soccer. Global programs, such as FIFA 11+ and BEP plyometric, provide players with hole strengthening and neuromuscular control. For these global programs, we may add muscle-specific exercises such as NHE and CA. Using structured devices is efficient in reducing IR, especially injury severity.

Finally, it goes without saying that prior to the exercise prescription, each player should have a specific evaluation and obtain an appropriately tailored program. In addition, it is important to target other risk factors, such as ethnicity and nutrition, which may explain the heterogeneity of the study findings. ^36^

## Conclusion

MI is the most frequent injury in football. It is responsible for long-term loss to play and the end of one’s career when it is severe. Coaches, doctors, and sports scientists have always tried to produce effective programs for MI and injury prevention in general.

In our study, we aimed to identify programs with proven efficacy in MI injury prevention. We reviewed the literature on PUBMED, COCHRANE, and GOOGLE SCHOLAR databases using PRISMA. We only included randomized controlled studies that were interested in determining the efficacy of an exercise or a complete program on reducing MI in professional and semi-professional soccer players, with more than 30 effective participants in each group.

Finally, we included seven articles published in journals of good quality according to the PEDRO scale in the last 10 years. The total number of participants was 3815 male players.

The program included FIFA 11+, BEP, stretching, and NHE. There was no significant difference in IR, but there was a significant difference in severe injury and, consequently, less time loss due to injury in the IG.

Finally, we can conclude that for MI prevention in football, we may use a completely structured program such as FIFA11+, for which we can add muscle-specific exercises such as NHE and CA exercises.

## Data Availability

All data underlying the results are available as part of the article and no additional source data are required. Repository name: Figshare PRISMA abstracts/PRISMA/PRISMA-Scr checklist and flowchart for: only severe injuries are effectively reduced by muscles’ injury prevention protocols in football players: A systematic review. Title: Efficacy of programs of muscle injuries prevention in Soccer https://figshare.com/s/b5b3d426ea978cde9797 License CCO Repository name: Figshare Title: Efficacy of programs of muscle injuries prevention in Soccer File: citation-export-muscle-injury.xls https://figshare.com/s/b5b3d426ea978cde9797 License CCO
