# Supplementary material for: Only severe Injuries are effectively reduced by muscles' injury prevention protocols in football players: A systematic review
Source: F1000Res. 2024 Dec 10;13:695. Originally published 2024 Jun 26. [Version 2] doi: 10.12688/f1000research.148925.2 (PMC11963548; doi:10.12688/f1000research.148925.2)
Supplement: Peer review history

# Reviewer report: Reviewer response for version 2

**DOI:** 10.5256/f1000research.175411.r347275

**Published:** 2024-12-16

**Authors:** Dhahbi Wissem

**Affiliations:** 1 Qatar Police College, Doha, Qatar

GENERAL COMMENTS

 The authors have successfully addressed all major concerns raised in the previous review. The manuscript has been significantly enhanced through:

 Strengths:

 - Comprehensive methodology with clear inclusion/exclusion criteria

 - Robust statistical analysis and appropriate interpretation

 - Well-structured presentation of findings

 - Thorough discussion of limitations including study timeline considerations

 - Strong practical implications for injury prevention in football

 The revisions have resulted in a manuscript that makes a valuable contribution to sports medicine literature, particularly regarding structured injury prevention programs.

 SPECIFIC COMMENTS

 Abstract:

 Page 1, Lines 15-18: Statistical results are now clearly presented with appropriate confidence intervals

 Page 2, Lines 12-14: Strong practical implications for implementation

 Introduction:

 Page 3, Lines 4-8: Epidemiological context well-supported with specific incidence rates

 Page 4, Lines 15-20: Clear rationale for investigating muscle injury prevention programs

 Methods:

 Page 5, Lines 10-12: Well-defined criteria for study inclusion and quality assessment

 Page 6, Lines 8-10: Transparent description of the review process

 Results:

 Page 9, Table 2: Clear presentation of findings with consistent statistical reporting

 Page 10, Lines 5-8: Thorough analysis of potential confounding factors

 Discussion:

 Page 12, Lines 8-12: Comprehensive discussion of clinical implications

 Page 13, Lines 15-20: Thoughtful consideration of study limitations

 Tables/Figures:

 - All tables now include clear abbreviations and explanatory notes

 - Figure 1 provides a clear overview of the study selection process

 - Statistical presentation is consistent throughout

Are the rationale for, and objectives of, the Systematic Review clearly stated?

Partly

Is the statistical analysis and its interpretation appropriate?

Partly

If this is a Living Systematic Review, is the ‘living’ method appropriate and is the search schedule clearly defined and justified? (‘Living Systematic Review’ or a variation of this term should be included in the title.)

Partly

Are sufficient details of the methods and analysis provided to allow replication by others?

Partly

Are the conclusions drawn adequately supported by the results presented in the review?

Yes

Reviewer Expertise:

Biomechanics, Sports Medicine, Rehabilitation and Sports injuries, Fitness and Exercise physiology

I confirm that I have read this submission and believe that I have an appropriate level of expertise to confirm that it is of an acceptable scientific standard.

# Reviewer report: Reviewer response for version 1

**DOI:** 10.5256/f1000research.163312.r339867

**Published:** 2024-11-18

**Authors:** Dhahbi Wissem

**Affiliations:** 1 Qatar Police College, Doha, Qatar

General Comments:

 This systematic review examines the effectiveness of muscle injury prevention protocols in professional football players. Overall, the manuscript addresses an important topic and follows proper systematic review methodology. However, there are several major and minor issues that need to be addressed to improve the quality and clarity of the work.

 Major Weaknesses:

 1. The literature search strategy appears limited, using only 3 databases and potentially missing relevant articles from other sources like SPORTDiscus or conference proceedings.

 2. Risk of bias assessment using only the PEDro scale provides limited evaluation of systematic review-specific bias domains recommended by Cochrane.

 3. The statistical analysis and synthesis of results lacks formal meta-analysis techniques or quantitative pooling of effect sizes across studies.

 4. Discussion section needs stronger critical analysis of the findings and clearer implications for clinical practice.

 Minor Weaknesses:

 1. Abstract could be more structured and detailed regarding specific outcomes.

 2. Some inconsistencies in reporting numerical results across tables and text.

 3. Reference citations need formatting updates in several places.

 4. Tables would benefit from additional explanatory footnotes.

 Specific Comments by Section:

 Abstract:

 Page 1:

 - Background section should specify the exact prevalence rates of muscle injuries

 - Methods section needs to state the specific databases searched with dates

 Line 12: Define "high level of evidence" explicitly

 Line 20: Report specific effect sizes and confidence intervals where available

 Introduction:

 Page 4:

 - Lines 2-5: Citation needed for injury incidence statistics

 - Lines 15-20: The rationale for focusing only on professional players needs stronger justification

 - Organization would benefit from clearer research question and objectives statements

 Methods:

 Page 5-6:

 - PRISMA checklist items should be explicitly addressed

 - Search strategy details incomplete - need full search strings for each database

 - Inclusion/exclusion criteria need more precise operational definitions

 - Statistical analysis plan lacks detail on handling heterogeneity

 Results:

 Pages 7-11:

 - PRISMA flow diagram missing some standard elements

 - Table 1 needs column explaining study design details

 - Effect sizes and precision estimates inconsistently reported across studies

 - Forest plots would help visualize key outcomes

 Discussion: 

 Pages 11-14:

 - Limited critical analysis of potential sources of bias

 - Inadequate discussion of heterogeneity across studies

 - Clinical implications need more specific recommendations

 - Future research directions should be expanded

 Insert the bellow reference:

 Iuliano, Enzo, et al. "Predictive ability of body mass parameter to estimate 4-6 repetition maximum of upper and lower limb muscles in soccer players." *Age (years)* 24 (2021): 4-66.[Ref 1] 

 Conclusion:

 Page 16: Conclusions should be more measured based on limitations identified

 Tables/Figures:

 - Table 1: Add study design details and risk of bias ratings

 - Table 2: Include confidence intervals for all effect estimates

 - Table 3: Clarify units of measurement

 - Table 4: Add statistical significance indicators

Are the rationale for, and objectives of, the Systematic Review clearly stated?

Partly

Is the statistical analysis and its interpretation appropriate?

Partly

If this is a Living Systematic Review, is the ‘living’ method appropriate and is the search schedule clearly defined and justified? (‘Living Systematic Review’ or a variation of this term should be included in the title.)

Partly

Are sufficient details of the methods and analysis provided to allow replication by others?

Partly

Are the conclusions drawn adequately supported by the results presented in the review?

Yes

Reviewer Expertise:

Biomechanics, Sports Medicine, Rehabilitation and Sports injuries, Fitness and Exercise physiology

I confirm that I have read this submission and believe that I have an appropriate level of expertise to confirm that it is of an acceptable scientific standard, however I have significant reservations, as outlined above.

## References

[1] : PREDICTIVE ABILITY OF BODY MASS PARAMETER TO ESTIMATE 4-6 REPETITION MAXIMUM OF UPPER AND LOWER LIMB MUSCLES IN SOCCER PLAYERS. *Acta kinesiologica* .2021; 10.51371/issn.1840-2976.2021.15.2.16 120-124 10.51371/issn.1840-2976.2021.15.2.16

# Response

**Published:** 2024-11-28

**Authors:** Rihab MONCER

**Affiliations:** physical and rehabilitation, University of Sousse, Faculty of medicine of Sousse, Sousse, Tunisia

We would like to express our sincere gratitude to the reviewer for their thoughtful and constructive feedback on our systematic review. We appreciate the time and effort taken to assess our manuscript. Below, we provide a detailed response to each of the comments raised, outlining the revisions we have made or plan to make in response.

**1. Comment 1:** The literature search appears limited, using only three databases and potentially missing relevant articles from other sources like SPORTDiscus or conference proceedings.

1.
  - **Response:** We acknowledge the reviewer’s concern regarding the scope of the literature search. Since the study is finished we have added as limitations the need to consider other databases.
2. **Comment 2:**
  - **Reviewer’s Comment:** The use of only the PEDro scale provides a limited evaluation of systematic review-specific bias domains.
  - **Response:** We used this scale for its specific items for physical interventions. We added in the discussion its limitations
3. **Discussion Section:**
  - **Reviewer’s Comment:** The Discussion section needs stronger critical analysis and clearer implications for clinical practice.
  - **Response:** We have revised the Discussion section to provide a more in-depth critical analysis of the findings, including a discussion of potential biases and study heterogeneity. Additionally, we have enhanced the clinical implications section by offering more specific recommendations for practice.

**Minor Weaknesses:**

1. **Abstract:**
  - **Reviewer’s Comment:** The Abstract could be more structured and detailed regarding specific outcomes.
  - **Response:** We have restructured the Abstract to include more specific details on the outcomes of the systematic review, such as effect sizes and confidence intervals, where available.
2. **Inconsistencies in Reporting Numerical Results:**
  - **Reviewer’s Comment:** Some inconsistencies in reporting numerical results across tables and text.
  - **Response:** We have thoroughly reviewed and corrected the numerical results across both the text and tables to ensure consistency.
3. **Reference Citations:**
  - **Reviewer’s Comment:** Reference citations need formatting updates.
  - **Response:** We have addressed all reference formatting issues and ensured consistency throughout the manuscript in accordance with the required citation style.
4. **Tables:**
  - **Reviewer’s Comment:** Tables would benefit from additional explanatory footnotes.
  - **Response:** We have added explanatory footnotes to all tables to improve clarity and assist with the interpretation of the data presented.

Once again, we would like to express our appreciation for the reviewer’s insightful comments, which have significantly contributed to improving the quality of our manuscript.

# Reviewer report: Reviewer response for version 1

**DOI:** 10.5256/f1000research.163312.r318645

**Published:** 2024-09-13

**Authors:** Ismail Dergaa

**Affiliations:** 1 Primary Health Care Corporation, Doha, Qatar

The manuscript titled "Only Severe Injuries Are Effectively Reduced by Muscle Injury Prevention Protocols in Football Players: A Systematic Review" presents an excellent systematic review for potential publication in F1000. This review offers valuable insights into the effectiveness of structured programs like FIFA 11+ for muscle injury prevention in football players. The methodological approach, adherence to PRISMA guidelines, and focus on high-evidence studies are commendable. However, a few minor revisions are necessary before the manuscript can be considered for indexing.

 Commentary 1: The introduction effectively sets the stage for the review, but it could benefit from a more in-depth exploration of the physiological mechanisms underlying muscle injuries in football. This additional context would provide a stronger rationale for the necessity of specific prevention programs. Also the introduction should be splitted to 3 main parts: (what is known, what is unknown the literature gaps, and finally the aim of the study which should be identical to the aim presented in the abstract).

 Commentary 2: The discussion addresses program effectiveness but could further explore reasons for variability in outcomes. Insights into demographic factors or differences in program implementation could provide valuable context for interpreting the results.

 Commentary 3: A significant limitation that should be addressed is the gap between the publication date of this review (2024) and the cutoff date for included articles (2022). This limitation should be explicitly stated and explained in the manuscript. Consider discussing potential implications of this time gap and how it might affect the review's findings and recommendations.

 Commentary 4: Throughout the manuscript, there is a tendency to use definitive language, particularly the term "conclude." It would be more appropriate to use more measured terms such as "suggest," "indicate," or "show associations" when presenting findings. This adjustment would better reflect the nature of the evidence and the inherent limitations of systematic reviews.

 Commentary 5: Table 1, the Impact factor of some journal is missing, why? please add it if no impact factor is associated to those journals please mention it, and please at the bottom of the table mention that the Impact factor mentioned is for which year. 

 Commentry 6: for all tables, add tables abbreviations. 

 Commentary 7: The conclusion, while concise, could benefit from emphasizing practical implications for coaches and practitioners. Consider adding specific recommendations for integrating these findings into training routines. Same goes for the conclusion of the abstract

Are the rationale for, and objectives of, the Systematic Review clearly stated?

Yes

Is the statistical analysis and its interpretation appropriate?

Yes

If this is a Living Systematic Review, is the ‘living’ method appropriate and is the search schedule clearly defined and justified? (‘Living Systematic Review’ or a variation of this term should be included in the title.)

Yes

Are sufficient details of the methods and analysis provided to allow replication by others?

Yes

Are the conclusions drawn adequately supported by the results presented in the review?

Partly

Reviewer Expertise:

Sports Medicine and Exercise sciences

I confirm that I have read this submission and believe that I have an appropriate level of expertise to confirm that it is of an acceptable scientific standard.

# Response

**Published:** 2024-11-28

**Authors:** Rihab MONCER

**Affiliations:** physical and rehabilitation, University of Sousse, Faculty of medicine of Sousse, Sousse, Tunisia

**Response to Reviewer’s Comments:**

 We would like to express our sincere gratitude to the reviewer for their positive and constructive feedback on our manuscript. We appreciate the time and effort taken to evaluate our work and provide thoughtful suggestions. Below, we provide a detailed response to each of the comments raised, along with the revisions made or planned in the manuscript.

**Commentary 1:**

**Reviewer’s Comment:** The introduction could benefit from a more in-depth exploration of the physiological mechanisms underlying muscle injuries in football. Additionally, the introduction should be split into three main parts: (1) what is known, (2) what is unknown (the literature gaps), and (3) the aim of the study, which should align with the aim presented in the abstract.

- **Response:** We agree with the reviewer’s suggestion to enhance the introduction by providing more context on the physiological mechanisms of muscle injuries in football. We have adjusted the introduction as asked

**Commentary 2:** **Reviewer’s Comment:** The discussion addresses program effectiveness but could further explore reasons for variability in outcomes, such as demographic factors or differences in program implementation.

- **Response:** We appreciate the reviewer’s insight. In response, we have expanded the discussion to address potential reasons for variability in the outcomes of muscle injury prevention programs.

**Commentary 3: **

- **Reviewer’s Comment: **A significant limitation is the gap between the publication date of this review (2024) and the cutoff date for included articles (2022). This limitation should be explicitly stated and explained in the manuscript, with a discussion of its potential implications.

- **Response:** We agree with the reviewer that this time gap is an important limitation. We have now explicitly stated this limitation in the manuscript and discussed the potential implications of including studies published before 2022. We explain that newer studies may provide additional insights into the effectiveness of muscle injury prevention protocols, which could affect the overall conclusions of the review.

**Commentary 4: **

- **Reviewer’s Comment:** Throughout the manuscript, the use of definitive language, particularly the term "conclude," may overstate the certainty of the findings. It would be more appropriate to use measured terms like "suggest," "indicate," or "show associations."
- **Response:** We appreciate the reviewer’s valuable feedback on the language used in the manuscript. We have carefully reviewed the manuscript and replaced terms such as “conclude”.

**Commentary 5: **

- **Reviewer’s Comment:** The impact factor of some journals is missing in Table 1. Please add it, or if no impact factor is associated with those journals, mention that explicitly. Additionally, please note at the bottom of the table the year the impact factor refers to.
- **Response:** We have updated Table 1 to include the impact factors for all journals listed.

**Commentary 6: **

- **Reviewer’s Comment:** For all tables, add table abbreviations.
- **Response:** We have added abbreviations to all tables, as suggested, to improve clarity and ensure the tables are easy to interpret. A key for abbreviations has been included at the bottom of each table.

**Commentary 7:**

**Reviewer’s Comment:** The conclusion could benefit from emphasizing practical implications for coaches and practitioners, including specific recommendations for integrating the findings into training routines. The same applies to the conclusion in the abstract.

**Response:** We have revised the conclusion to emphasize the practical implications for coaches and practitioners. Specific recommendations for integrating muscle injury prevention protocols into training routines have been added. We have also updated the conclusion in the abstract to reflect these practical recommendations.

 Once again, we thank the reviewer for their insightful comments, which have greatly contributed to improving the quality of our manuscript. We believe the revisions we have made address all the points raised and enhance the clarity and relevance of our review. We look forward to your further feedback.
